# Supplementary material for: The Promotion of Migration and Myogenic Differentiation in Skeletal Muscle Cells by Quercetin and Underlying Mechanisms
Source: Nutrients. 2022 Oct 2;14(19):4106. doi: 10.3390/nu14194106 (PMC9572605; doi:10.3390/nu14194106)
Supplement: Supplementary file 1 [file nutrients-14-04106-s001.zip › nutrients-1927030-supplementary.pdf]

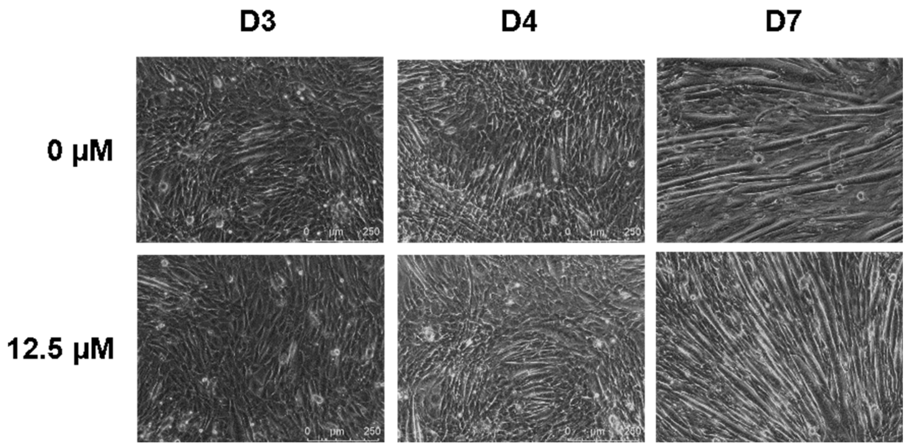

Figure S1. Microscope images of C2C12 cells change morphology after 3,4,7 days. Compare cell morphology changing between the control group and quercetin (12.5 μM) treatment.

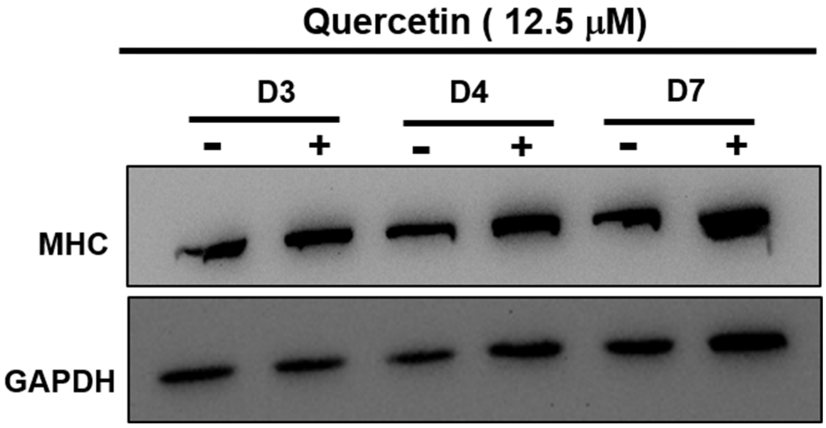

Figure S2. MHC protein expression level related to quercetin (12.5 μM) and control group after 3,4,7 days of treatment. Western blot analysis was used to determine protein expression of MHC.
